# Influence of air pollutants on pneumonia hospitalizations among children in a town in the Brazilian Legal Amazon region: a time series study

**DOI:** 10.1590/1516-3180.2019.0456.R1.09122019

**Published:** 2020-06-01

**Authors:** Danila Pequeno Santana, Viviane Martins Santos, Ageo Mário Cândido da Silva, Walkiria Shimoya-Bittencourt

**Affiliations:** I RN. Nurse and Master’s Student, Postgraduate Program on Environment and Health, Universidade de Cuiabá (UNIC), Cuiabá (MT), Brazil.; II PT, MSc, PhD. Physiotherapist, Universidade Federal de Mato Grosso (UFMT), Hospital Universitário Júlio Müller, Cuiabá (MT), Brazil.; III PT, PhD. Pharmacist-Biochemist, Postgraduate Program on Environment and Health, Universidade de Cuiabá (UNIC), Cuiabá (MT), Brazil.; IV PT, PhD. Physiotherapist, Postgraduate Program on Environment and Health, Universidade de Cuiabá (UNIC), Cuiabá (MT), Brazil.

**Keywords:** Particulate matter, Pneumonia, Child, Air pollution, Children, Amazonian ecosystem, Agricultural pollution

## Abstract

**BACKGROUND:**

Exposure to particulate material produced as a result of increased agricultural activity may increase the number of pneumonia hospitalizations among children. We hope to contribute to the knowledge base through highlighting the environmental mechanisms involved in this outcome and optimizing pollutant control policies.

**OBJECTIVES:**

To investigate the association between pneumonia hospitalizations among children and presence of environmental pollutants in a town in the Brazilian Legal Amazon region.

**DESIGN AND SETTING:**

Time series study conducted in the town of Tangará da Serra, Mato Grosso (MT), Brazil.

**METHODS:**

A total of 158 children aged 0 to 10 years participated in the study. Data on environmental variables and pollutants were extracted daily through the Coupled Chemistry-Aerosol-Tracer Transport model coupled to Brazilian Regional Atmospheric Modeling System (CCATT-BRAMS). Meteorological data were provided by the Weather Forecasting and Climate Studies Center (CPTEC).

**RESULTS:**

There was greater frequency of pneumonia hospitalizations in the months of transition between the rainy and dry seasons, with a prevalence ratio 2.4 times higher than in other periods. For environmental pollutants, there was a significant positive correlation between particulate matter (PM_2.5_) and pneumonia hospitalizations (correlation 0.11), with more admissions on the days when PM_2.5_ levels were highest (averages of 6.6 µg/m^3^ when there were no admissions and 13.11 µg/m^3^ on days with two or more admissions).

**CONCLUSIONS:**

The higher the PM_2.5_ level was, the greater the frequency of hospitalizations also was. Children living in peripheral areas had higher prevalence of pneumonia hospitalizations in the dry period than those who were living in the town center.

## INTRODUCTION

Air pollution is gaining increasing importance within the environmental scenario because it causes great risks to health, with higher risk of death and respiratory diseases among children.^1^^,^^2^ In 2016, one out of every nine deaths among children was attributed to the effects of pollution, with a total of 7 million deaths worldwide.^3^

Particulate matter (PM) is a mixture of solid and liquid components formed by a variety of compounds that depend on the emission source. These fine and ultra-fine particles can reach the alveoli, where they are phagocytized by macrophages and neutrophils that release inflammatory mediators and may cause irritation to the eyes, throat and lungs. According to the World Health Organization (WHO), the mean limit of acceptability of exposure for particulate matter of size smaller than 2.5 µm (PM_2.5_) is a concentration of 25 µg/m^3^ for 24 hours.^4^ Several mechanisms are involved in the respiratory disease caused by particulate matter, especially induction of pulmonary oxidative stress. This leads to overproduction of oxidative reaction, thus damaging the deoxyribonucleic acid (DNA) and inducing inflammatory lesions and epigenetic disorders, thereby contributing to the development of diseases such pneumonia.^5^^,^^6^^,^^7^^,^^8^

Carbon monoxide (CO) is a systemic asphyxiant that induces depression of the central nervous system that at acute levels can cause death because it has affinity for hemoglobin that is 200 times higher than that of oxygen. In situations of chronic poisoning, slow hypoxia may develop and this may lead to permanent sequelae.^9^ National Environmental Council (Conselho Nacional do Meio Ambiente, CONAMA) resolution no. 3 of 1990 sets the parameters for air quality as an average concentration of 10,000 mg/m^3^ (9 ppm) over an eight-hour period and an average concentration of 40,000 mg/m^3^ (35 ppm) over a one-hour period that is not to be exceeded more than once a year.^10^

## OBJECTIVE

The objective of this study was to investigate the influence of pollutants on pneumonia hospitalizations among children in a town in the Brazilian Legal Amazon region.

## METHODS

A time series study was conducted among children living in the town of Tangará da Serra, Mato Grosso (MT), Brazil, from August 1, 2017, to July 31, 2018. It was approved by the local research ethics committee under opinion no. 2.325.965 on October 10, 2017.

Tangará da Serra is located in a region with plenty of agricultural activities and is in the region of the arc of deforestation of the Brazilian Amazon region. It has well-defined seasonal periods, with a rainy season (November to March), a transition period (April and May) and a dry season (June to October). In 2005, this town presented the highest rate of hospitalizations due to respiratory diseases in its state (Mato Grosso, MT), among children younger than 15 years of age, and pneumonia was the leading cause of hospitalization (90.7%).^11^ The high rate of hospitalizations, along with the location of the town, are particularly relevant.

Convenience sampling was performed among the children hospitalized at a public hospital who presented a clinical diagnosis of pneumonia that had been confirmed by a pediatrician. This diagnosis was verified by the researcher in loco with the pediatrician, though radiographs, laboratory tests and clinical examination. This sampling was done according to convenience because we investigated all the children hospitalized over a one-year period to confirm the diagnoses, identify geographical data relating to their homes and ascertain their health histories.

The study included children aged 0 to 10 years who were living in the town of Tangará da Serra (MT), in either its urban area or its rural area. Children were excluded from the study if they had pneumonia with associated comorbidities such as chronic diseases of the respiratory system; autoimmune, neurological or immunosuppressive diseases; prolonged use of corticosteroids; oncological treatment; long-term bedridden state; or malnourishment.

Health history and demographic data were collected from the parents/guardians during their children’s hospitalization, through a questionnaire that the authors created. Nutritional characteristics were assessed using the body mass index (BMI). Data were collected on a regular basis from the patients’ medical records through an instrument that the authors developed.

The cases were grouped according to the neighborhood in which the children lived. These neighborhoods were analyzed regarding their proximity to the downtown region and to the peripheral areas (i.e. the areas closest to the highway and to agricultural activities).

Data on pollutants were collected daily from the Coupled Chemistry Aerosol and Tracer Transport model for the Brazilian developments on the Regional Atmospheric Modelling System (CCATT-BRAMS), which is one of the strategies commonly used in research carried out in regions that do not have monitoring stations, such as the state of Mato Grosso. CCATT-BRAMS is a reliable mathematical model that is used to make emission estimates. These data are made available daily in Grid Analysis and Display System (GRADS) binary format files corresponding to South America. A specific point for these estimates was determined through the geographic coordinates of the municipality (latitude: -14.6279; longitude: -57.507). Estimates for the pollutants CO and particulate matter (PM_2.5_) were selected because of the lack of complete data on other pollutants.

Climate data were provided by the Weather Forecasting and Climate Studies Center of the National Institute for Space Research (Centro de Previsão de Tempo e Estudos Climáticos, CPTEC) through the Brazilian National Institute of Meteorology (Instituto Nacional de Metereologia, INMET) automatic weather station that is installed at the Mato Grosso State University Campus. The data were provided in the form of Excel spreadsheets. The climate variables collected were relative air humidity, environmental temperature, wind speed and precipitation.

To seek a correlation between environmental variables and pneumonia hospitalizations, univariate analysis was performed to obtain central trend and dispersion measurements. Bivariate analysis was carried out through the use of Spearman correlation analysis for nonparametric data. Multiple linear regression was performed to identify which variables were predictors for pneumonia hospitalization. Also, multivariate analysis was performed using the Kruskal-Wallis model to estimate the relationship between frequency of pneumonia hospitalization and exposure to environmental variables. The dependent variables were hospitalizations for pneumonia and CO and PM_2.5_ levels and the independent variables were the climate variables.

Associations were assessed between variables that correlated with the cases of hospitalization due to pneumonia, on the day of hospitalization. We separated the daily hospitalizations into three categories: days without hospitalizations, days with one hospitalization and days with two or more hospitalizations. To these, we applied the Kruskal-Wallis test and one-way analysis of variance (ANOVA). The chi-square test was used to evaluate the number of daily hospitalizations per seasonal period (drought, transition and rain).

For all analyses, the significance level was set at P = 0.05, and the Epi-Info 6.04 and SPSS version 20.0 software was used.

## RESULTS

A total of 158 children participated in the study; 121 (76.6%) had a diagnosis of bronchopneumonia; 82 (51.9%) were males and 97 (61.4%) had brown skin color. Regarding their health history, 153 (96.8%) of the children were born at term and 109 (69%) had received all vaccines, with three doses of pneumococcal vaccine (Pneumo 10). There were 95 children (60.1%) aged between one and five years old (Table 1).

**Table 1.** Clinical and demographic characteristics of children aged 0 to 10 years admitted to a public hospital in the town of Tangará da Serra (MT), 2017- 2018

Characteristics	n = 158 (%)
Diagnosis	
Bronchopneumonia Pneumonia	121 (76.6) 37 (23.4)
Sex	
Male Female	82 (51.9) 76 (48.1)
Color	
Brown Black White	97 (61.4) 56 (35.5) 5 (3.1)
Age	
Younger than 1 year 1 to 5 years 6 to 10 years	44 (27.8) 95 (60.1) 19 (12.1)
Prematurity	
Yes No	5 (3.2) 153 (96.8)
Vaccination with Pneumo 10	
No vaccine Ongoing vaccination Completed vaccination Late vaccination	4 (2.5) 39 (24.7) 109 (69) 6 (3.8)

Regarding the distribution of cases in the city, it was observed that most were concentrated on the periphery, especially in neighborhoods near the highway and agricultural activities. Thus, there were 61 cases (38.6%) in peripheral neighborhoods, 16 cases (10.1 %) in the countryside and only 4 cases in the city center (2.5%).

In the analysis on the correlations of environmental variables, air pollutants and hospitalization for pneumonia, there was a significant positive correlation between the number of pneumonia hospitalizations and PM_2.5_ levels, and a negative correlation between the number of hospitalizations and the relative humidity and rainfall. Furthermore, it was found that CO levels showed significant positive correlations with PM_2.5_ levels and temperature, and significant negative correlations with humidity and wind speed. PM_2.5_ levels presented significant negative correlations with humidity and rain. These results are shown in Table 2.

**Table 2.** Analysis of correlations between environmental variables and pneumonia hospitalizations. Tangará da Serra (MT), 2017-2018

	PNMHosp	CO	PM_2.5_	T	RH	Wind	Rain
PNMHosp	1						
CO	-0.51	1					
PM_2.5_	0.11*	0.41*	1				
T	0.07	0.34*	0.03	1			
RH	-0.10*	-0.10*	-0.38*	-0.52*	1		
Wind	-0.26	-0.20*	-0.16*	-0.41*	0.38*	1	
Rain	-0.13*	0.15*	-0.38*	-0.20*	0.64*	0.23*	1

*Significant correlation for P < 0.05.

PNMHosp = pneumonia hospitalizations; CO = carbon monoxide; PM_2.5_ = particulate matter; T = temperature; RH = relative air humidity.

Analysis on the behavior of pollutants between different seasonal periods showed that CO levels were higher during the rainy season, despite maintaining an average of 0.1 ppm, although there was a significant negative correlation with humidity. PM_2.5_ levels were higher in the dry season, with an average of 10.7 µg/m^3^.

Among the climate variables (Table 3), temperature (T) was higher in the rainy season with an average of 25 °C. Relative humidity (RH) was higher in the rainy season (80.9%). Wind speed was higher in the rain season and transition period, with a speed of 2.1 m/s in both of these periods. Radiation was greater in the dry season, with an average of 765.6 kJ/m^2^.

**Table 3.** Means for pollutants and climate variables in the dry, rainy and transition periods. Tangará da Serra (MT), 2017-2018

Variables	Dry season	Rainy season	Transition period
PM_2.5_			
Mean (SD)	10.7 (7.9)	4.2 (5.6)	7.5 (10.4)
Minimum-maximum	1.9-54.5	0.1-32.2	1-66.8
CO			
Mean (SD)	0.1 (0.1)	0.1 (0.1)	0.1 (0.03)
Minimum-maximum	0.1-0.4	0.1-0.5	0.05-0.2
T			
Mean (SD)	24.5 (3.5)	25 (1.2)	24.1 (1.9)
Minimum-maximum	12.7-29.7	20.6-28.4	16.5-26.2
RH			
Mean (SD)	61.3 (16.4)	80.9 (4.7)	79.7 (5.2)
Minimum-maximum	30-96.5	70.4-96.1	67.2-91.8
Wind			
Mean (SD)	1.3 (1.3)	2.1 (0.6)	2.1 (0.6)
Minimum-maximum	0-5.4	0.9-3.8	1.1-3.6
Rain			
Mean (SD)	0.02 (0.1)	10.3 (19)	3.4 (8.2)
Minimum-maximum	0-0.6	0-159	0-48.8
Radiation			
Mean (SD)	765.6 (190.2)	18.3 (4.6)	17 (3.4)
Minimum-maximum	196.4-1286	2.2-27.8	7-22.5

CO: ppm; PM_2.5_: µg/m^3^; T: °C; RH: %; wind: m/s; rain: mm; radiation: kJ/m^3^.

PM_2.5_ = particulate matter; CO = carbon monoxide; T = temperature; RH = relative air humidity; SD = standard deviation.

After multiple linear regression, it was found that PM_2.5_ level, RH and period of the year were predictors for pneumonia hospitalization among these children, as shown in Table 4.

**Table 4.** Predictors for pneumonia hospitalization among children that were found to be statistically significant through multiple linear regression

Variables	Coefficients	Standard error	t	P
Period of the year	0.182	0.039	4.634	< 0.001
PM_2.5_ µg/m^3^	0.009	0.002	3.933	< 0.001
RH (%)	-0.008	0.002	-4.152	< 0.001

CO (carbon monoxide): adjustment variable.

PM_2.5_ = particulate matter; RH = relative air humidity.

For better understanding of the positive correlation between PM_2.5_ level and pneumonia hospitalizations, the distribution of daily hospitalizations was divided into three categories: no hospitalization, one hospital admission and two or more hospital admissions per day. The Kruskal-Wallis test showed mean values for PM_2.5_ of 6.6 µg/m^3^ on the days when there was no hospitalization, 7.39 µg/m^3^ on the days when there was one hospital admission and 13.11 µg/m^3^ on the days when there were two or more hospital admissions. There were higher numbers of hospitalizations as the PM_2.5_ level increased. Regarding seasonality, there was a significant difference between the groups, with an association between a greater number of hospitalizations per day and the transition period, with a prevalence ratio 2.4 times higher in this period than in other periods.

**Figure 1.**
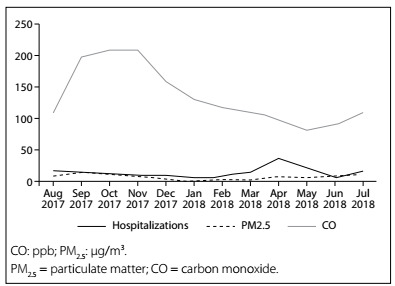
Pneumonia hospitalizations among children aged 0 to 10 years and levels of PM_2.5_ and CO according to the period of the year, in Tangará da Serra (MT), 2017-2018.

Comparing the period of the year and the numbers of pneumonia hospitalizations among the children (Figure 1), there were higher numbers of hospitalizations due to pneumonia/bronchopneumonia in the months of August and September (dry season), lower numbers between November and February (rainy season) and a peak in the numbers of hospitalizations in the months of April and May (transition period), in 2018.

## DISCUSSION

There was a higher number of pneumonia hospitalizations among children aged 0 to 5 years, with predominance among males. This had previously been seen in other studies, which reported that this difference between males and females was due to anatomical reasons. This was ascribed, for example, to immunological immaturity and reduced airway caliber, along with greater exposure to risk factors among boys at this age.^4^^,^^11^^,^^12^

The findings showed that there was a significant positive correlation of PM_2.5_ levels with pneumonia hospitalizations. This had also been reported in studies conducted in Brazil by Machin and Nascimento,^13^ in the city of Cuiabá, and by Negrisoli and Nascimento,^14^ in the city of Sorocaba. These authors reported that hospitalization occurred four days after exposure. This was also shown in a systematic review in the state of São Paulo, in which an association between presence of particulate matter of size 10 µm (PM_10_) and respiratory tract diseases in children was found.^15^

This association can be explained by several factors, such as presence of oxidative stress, structural damage and immune system disorders associated with carbon compounds and biomass burning. These lead to inflammation and harm to health, especially among children younger than 10 years.^16^^,^^17^^,^^18^ On the other hand, Tuan, Venâncio and Nascimento^19^ did not identify any correlation between presence of PM_10_ and pneumonia hospitalization among children under five years of age. The reason for their findings was that there was little emission of PM during the study period and few hospitalizations, which may be indicative of a dose-response effect.

CO levels did not show any significant positive correlation with the number of pneumonia hospitalizations. This finding differed from those of studies that correlated CO levels with car traffic. For example, in a study conducted among schoolchildren in the city of Quito, Ecuador, it was reported that there was a substantial decline in CO levels after a five-year program of vehicle emission control, together with a reduction in the incidence of respiratory diseases.^20^ In a retrospective cohort study among children up to two years of age in Atlanta, United States, the PM_2.5_, nitrogen oxide (NO_X_) and primary CO levels from vehicle traffic showed an association with the presence of otitis media and bronchiolitis.^21^

In our study, it appears that pollution caused by agricultural activity is higher than pollution resulting from vehicle traffic, since business activity in Tangará da Serra is essentially agricultural and its car fleet is not large, which also explains the low rates of CO emission in the study period.

There are some differences in the literature regarding relative air humidity. In a study by Kim et al.,^4^ it was found that there were differences among the geographical regions studied. There was a positive correlation in some regions but a negative one in others, which the authors attributed to local environmental factors. Ho et al.^22^ evaluated climatic effects on the population aged 0 to 16 years living in Ho Chi Minh City, Vietnam. The authors reported that higher incidence of pneumonia hospitalizations was associated with higher humidity and precipitation. In a study by Andrade Filho et al., conducted in Manaus, Brazil, there was a significant positive association (R = 0.126) between greater numbers of hospitalizations due to respiratory diseases among children and higher relative humidity in the Pearson correlation analysis. Moreover, high humidity explained 84% of the hospitalizations.^23^

Regarding the frequency of hospitalization in different seasonal periods, Santos et al.^24^ investigated the population aged 0 to 5 years in the city of Rondonópolis, Brazil. They reported that there were greater numbers of hospitalizations due to pneumonia in the months of April and May. They attributed this to the sudden change of temperature that occurs in these months.

Ignotti et al.^25^ found that hospitalizations due to respiratory diseases among children aged 0 to 5 years in Tangará da Serra peaked at the end of the rainy season (April). In analyzing pneumonia hospitalizations in the same town, Rosa et al.^11^ found a reduction in the frequency of hospitalizations during the rainy months, followed by an increase in the number of cases in March and subsequently a 10% increase in hospitalizations during the months of drought.

There was a higher prevalence rate of pneumonia among children who were living in peripheral areas, during the drought period. Pinto, Maggi and Alves^26^ reported that children younger than five years who were living in rural areas of the state of Pernambuco had twice as much risk of pleural involvement in pneumonia as did those who were living in urban areas. This finding correlated with worse conditions of sanitation and lower income. However, in a study on a population aged 2 to 18 years in the state of Georgia, United States, Strikland et al.^27^ did not find any association between levels of urbanicity and pneumonia hospitalizations. They attributed their finding to the limitation of their study of not characterizing the composition of PM_2.5_ in each area, since its composition differs between urban and rural areas.

The state of Mato Grosso has strong presence of agribusiness, with intense agricultural activity in the months with peaks of pneumonia hospitalization among children. These activities include controlling of cotton crop pests, herbicide application on sugar cane, preparation and planting and fertilization of soil for corn and soybean crops.^28^

In a study conducted in California, Ganesh and Smith^29^ highlighted the strong influence of anthropogenic actions on PM_2.5_ emissions resulting from agricultural pollution, such as cultivation, harvesting and vehicle traffic. Their findings showed that mitigation activities may benefit the health of populations. In a study conducted in China, it was found that a reduction in the number of farms in a region may reduce the regular levels of pollution from agricultural sources.^30^ In a study conducted in Turkey, there was harm to the respiratory tract after use of pesticides in agriculture.^31^

Importantly, the effects of PM_2.5_ on human health depend on its composition. In a systematic review in which the aim was to identify the properties of PM in the Amazon biome, its predominant characteristic was found to be high concentrations of biogenic elements during the rainy season and anthropogenic elements during the dry season.^32^ In another study in Tangará da Serra, there was predominance of anthropogenic emissions over biogenic emissions.^33^ These findings, which correlated with higher prevalence of pneumonia among children living in peripheral areas, may indicate that agricultural activities were having an influence. In the transition months, there was greater wind speed, which may have dispersed the emissions produced by agricultural activities that were closer to the outskirts of the town, which may explain the increase in hospitalizations.

Policies for controlling pollutant emissions are paramount for reducing the damage to the health of vulnerable populations. This was highlighted in a systematic review that was carried out to ascertain whether proximity to pollutants in the environment could cause adverse health outcomes. It was shown that populations living close to environmental risks seemed to be more likely to have adverse health outcomes, although this did not necessarily mean exposure at the individual level.^34^

The limitations of the present study included its use of secondary data to obtain the environmental variables with the CCATT-BRAMS system, even though this is recognized to be a reliable mathematical model for estimates on emissions, along with its use of CPTEC data. In addition, the subjects were recruited through convenience sampling at the only public hospital in the town. The sample was only of small size.

## CONCLUSIONS

The higher the levels of PM2.5 were, the greater the frequency of hospitalizations also was. Children living in peripheral areas showed higher prevalence of pneumonia hospitalization during the dry season than did those who were living in the town center. Controlling and monitoring of air pollutant emissions, along with recognition of the type of particulate matter emitted in each region, can significantly help reduce unfavorable outcomes. In particular, this is enabled through recognition of anthropogenic influences and implementation of measures for mitigation of the impact of agricultural activities.
